# A Rare Cause of Acute Kidney Injury: Primary Renal Lymphoma in a Patient with Human Immunodeficiency Virus

**DOI:** 10.1155/2018/8425985

**Published:** 2018-08-13

**Authors:** Ruslinda Mustafar, Lydia Kamaruzaman, Beh Hui Chien, Azyani Yahaya, Noor'Ain Mohd Nasir, Rozita Mohd, Rizna Cader, Kong Wei Yen

**Affiliations:** ^1^Department of Medicine, Nephrology Unit, Universiti Kebangsaan Malaysia Medical Centre (UKMMC), Kuala Lumpur, Malaysia; ^2^Department of Medicine, Universiti Kebangsaan Malaysia Medical Centre (UKMMC), Kuala Lumpur, Malaysia; ^3^Department of Pathology, Universiti Kebangsaan Malaysia Medical Centre (UKMMC), Kuala Lumpur, Malaysia

## Abstract

We reported a case of primary renal lymphoma (PRL) presented with non-oliguric acute kidney injury and bilateral kidney infiltrates in an individual with human immunodeficiency virus (HIV) disease. Acute kidney injury secondary to lymphoma infiltrates is very rare (less than 1% of hematological malignancy). A 37-year-old gentleman with underlying human immunodeficiency virus (HIV) disease was on combined antiretroviral therapy since diagnosis. He presented to our center with uremic symptoms and gross hematuria. Clinically, bilateral kidneys massively enlarged and were ballotable. Blood investigations showed hemoglobin of 3.7 g/L, urea of 65.6 mmol/L, and serum creatinine of 1630 *µ*mol/L with hyperkalemia and metabolic acidosis. An urgent hemodialysis was initiated, and he was dependent on regular hemodialysis subsequently. Computed tomography renal scan showed diffuse nonenhancing hypodense lesion in both renal parenchyma. Diagnosis of diffuse large B cell lymphoma with germinal center type, CD20 positive, and proliferative index 95% was confirmed via renal biopsy, and there was no bone marrow infiltrates. Unfortunately, the patient succumbs prior to initiation of chemotherapy.

## 1. Introduction

Primary renal lymphoma (PRL) is one of the rare extranodal non-Hodgkin lymphoma. It is defined as non-Hodgkin lymphoma arising in renal parenchyma and not invasion from neighboring lymphomatous lesion. The first case was reported by Coggins in 1980 [1]. PRL attributes to less than 1% of all renal lesions, and bilateral kidney involvements are seen in 10–20% of the cases [2]. There is still no clinical trial to establish a diagnostic criteria or standard treatment for PRL due to shortage of cases.

In adult age from 18 to 50 years old, PRL usually presented with abdominal and flank pain while weight loss and gross hematuria are seen more commonly in adults more than 50 years old [3]. Kidney involvement in lymphoma can be presented with acute kidney injury (AKI), acute tubular necrosis (ATN), renovascular disease, parenchymal infiltration, obstructive uropathy, glomerulopathies, electrolyte, and acid-base imbalance [4]. Kidney injury may be due to underlying malignancy or secondary to complication of therapy.

## 2. Case Scenario

A 37-year-old gentleman with underlying human immunodeficiency virus (HIV) diagnosed in June 2015 was under infectious disease clinic follow-up from another center. He was started on combined antiretroviral therapy (tenofovir-emtricitabine and efavirenz) since diagnosis but defaulted treatment a month prior to his admission to our center. His CD4 count upon diagnosis was 20 cells/microliter and serum creatinine of 108 *µ*mol/L. After 12 months of treatment, his HIV viral load was less than 20 cells/L, and CD4 increased to 178 cells/microliter and serum creatinine of 193 *µ*mol/L. During his follow-up in February 2017, serum creatinine rose markedly to 1051 *µ*mol/L but remained asymptomatic; he was advised for admission and further investigation, but the patient refused due to some family issues.

He presented to our center in April 2017 with vomiting and gross hematuria for one week associated with abdominal pain and distention. He was also symptomatic of anemia. He had loss of weight and loss of appetite. He had good urine output (more than one liter in a day) and no frothy urine. He did not take any nephrotoxic drugs, that is, herbal supplementation or nonsteroidal anti-inflammatory drugs. Clinically, he was pale, and bilateral kidneys were ballotable and massively enlarged with an irregular surface and hard in consistency. There were no hepatomegaly and splenomegaly. Peripheral lymph nodes were not palpable as well.

**Figure 1:**
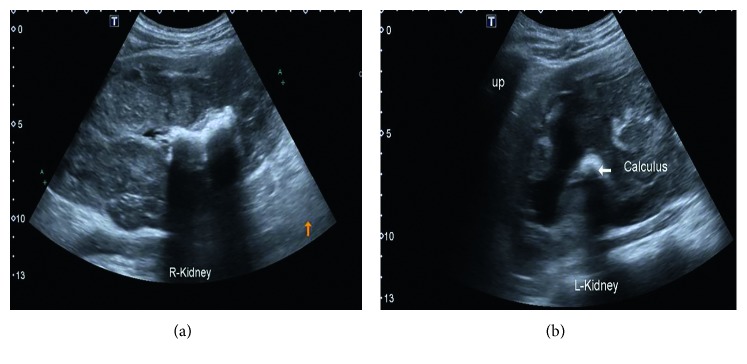
Ultrasound of the abdomen showed heterogeneous mass involving the right kidney and lower pole of the left kidney extending to the retroperitoneal space. The right kidney measured 17.1 cm, and the left kidney measured 14.6 cm. Both kidneys had loss of normal configuration. There was presence of staghorn calculi within the right kidney and calculus at the upper pole of the left kidney.

**Figure 2:**
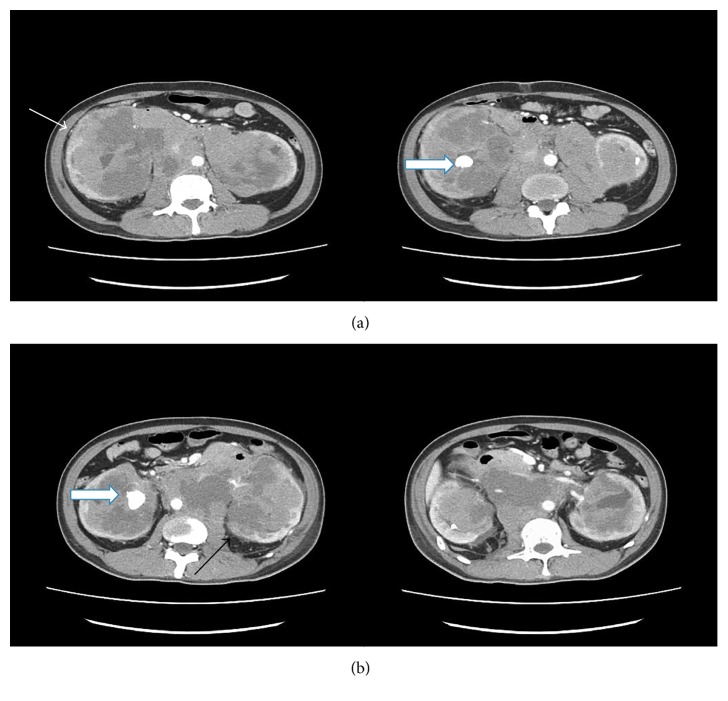
Four-phase renal computed tomography (CT) showed both kidneys were enlarged with the right kidney measuring 18 cm (white arrow) and the left kidney 14 cm (black arrow). There was a concomitant large renal calculus bilaterally, with the largest on the right measuring 1.5 cm × 2.0 cm × 2.2 cm (thick white arrow) and the left measuring 2.2 cm × 1.7 cm × 1.0 cm in the renal pelvis. Smaller renal calculi are seen in the rest of the pelvicalyceal systems.

Blood investigations showed hemoglobin of 3.7 g/L (normochromic and normocytic), total white count of 7.6 × 10^9^/L, lymphocyte count of 1.7 × 10^9^/L, and neutrophil count of 5.4 × 10^9^/L and urea of 65.6 mmol/L, serum creatinine of 1630 *µ*mol/L, potassium of 5.4 mmol/L, sodium of 132 mmol/L, hyperphosphatemia of 3.70 mmol/L, and corrected serum calcium of 2.48 mmol/L. Venous blood gas showed metabolic acidosis with pH of 7.18 and bicarbonate of 11 mmol/L. Urinalysis showed urine protein 2 + (0.75 g/L) and blood 3 + (250 *μ*/L). An internal jugular double-lumen catheter was inserted, and hemodialysis was initiated. Ultrasound abdomen (Figure 1) and 4-phase renal computed tomography (CT) (Figure 2) were done.

**Figure 3:**
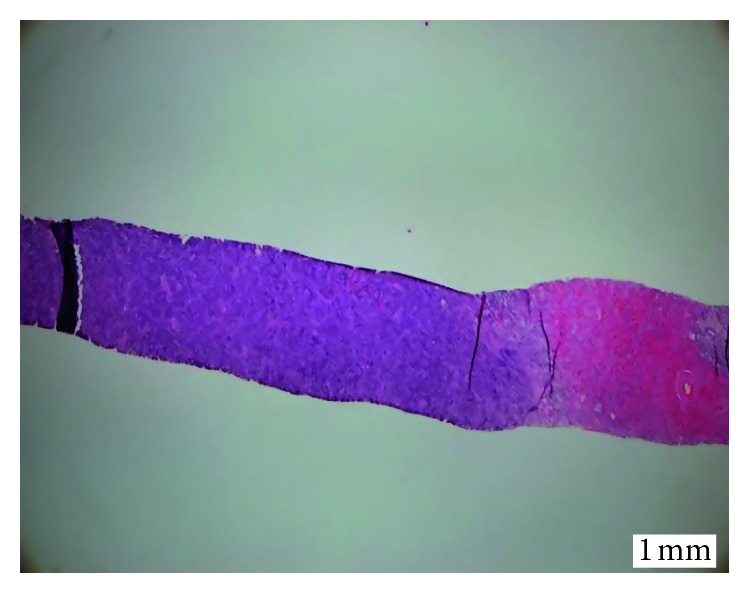
Three strips of tissue infiltrated by diffuse sheets of malignant cells (hematoxylin-eosin ×40). There were no normal glomeruli seen.

**Figure 4:**
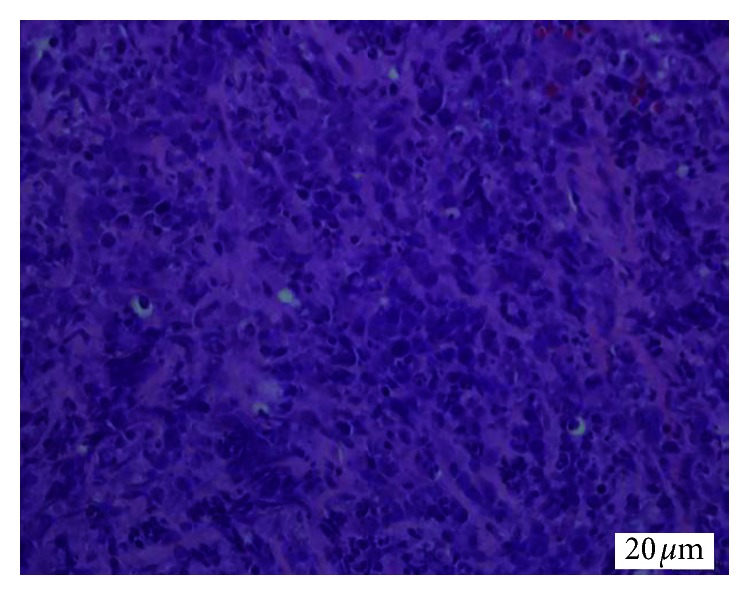
Malignant lymphoid cells exhibit marked pleomorphism with hyperchromatism. They were large with high nuclear to cytoplasmic ratio (hematoxylin-eosin ×400).

**Figure 5:**
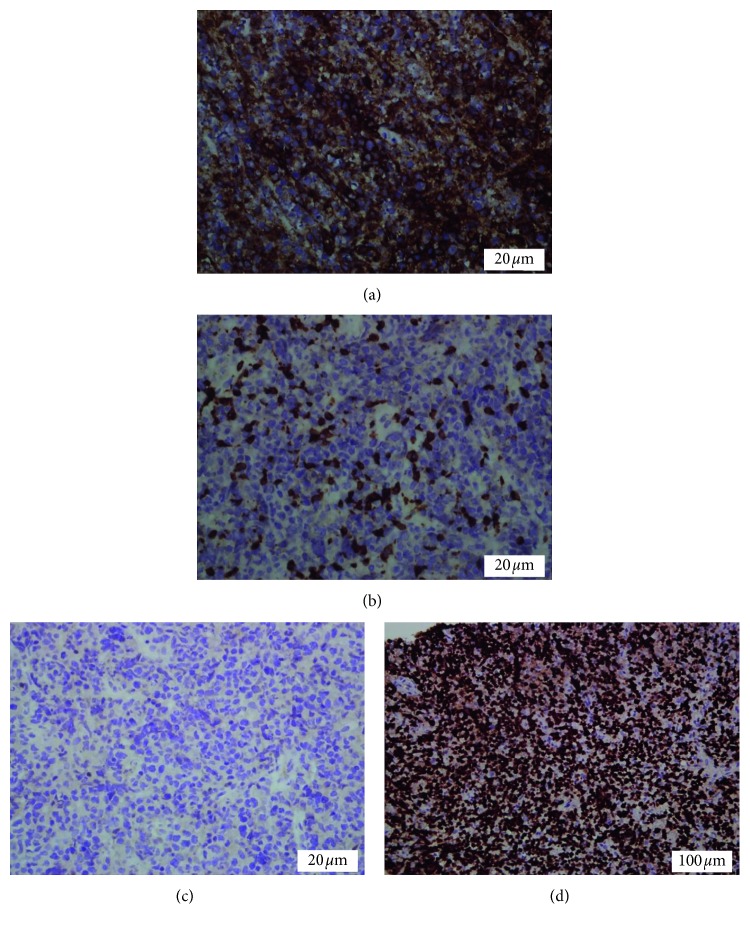
Immunohistochemical studies showed the malignant cells positive for CD20 (a), CD10, and BCL6. They are negative for CD3 (b) and MUM-1 (c), and Ki-67 (proliferative index) is 95% (d).

The ultrasound showed enlarged bilateral kidneys with calculus, and his 4-phase renal CT showed diffuse nonenhancing hypodense lesion in both renal parenchyma. The lesion at the right kidney extends medially to the paraaortic region, forming another hypodense mass component, measuring 6.4 cm × 8.1 cm × 11 cm. The mass is displacing the branches of the aorta, duodenum, and pancreas anteriorly. Subcentimetre nodes were seen in the mesenteric and inguinal region. In view of definite diagnosis cannot be made via imaging, a renal biopsy was arranged. Histopathology and immunochemistry result of left renal tissue showed diffuse large B cell lymphoma with the germinal center type (Figures 3–5).

A bone marrow aspirate and trephine (BMAT) was done and showed no bone marrow infiltration. Positron emission tomography CT scan (PET-CT) concluded extensive malignant lesion in the upper abdomen involving the kidneys, stomach, and pancreas. The liver, spleen, lungs, and brain have normal uptake. There were malignant lesions also found in the right cervical node, left tonsils, and posterior chest wall. Some small bone metastasis was also detected.

His repeat CD4 count was 84 cells/microliter, and the viral load was 6974 copies per ml. He was restarted on antiretroviral treatment: oral Kivexa (abacavir/lamivudine) 1 tab daily and oral efavirenz 600 mg daily, added oral trimethoprim and sulfamethoxazole (Bactrim) 2 tablets daily as prophylaxis prior to starting chemotherapy. He underwent regular hemodialysis and was transfused with packed red blood cell. He was planned for standard treatment of rituximab-cyclophosphamide, hydroxydaunorubicin, Oncovin, and prednisone (R-CHOP), but the patient developed upper gastrointestinal bleed and succumbed to death before starting the chemotherapy.

## 3. Discussion

The clinical presentation of PRL can be similar to other renal pathologies such as renal cell carcinoma, renal abscess, renal tuberculosis, or secondary metastasis. The symptoms range from hematuria, acute or chronic kidney injury, and loin pain or weight loss. Imaging findings of poorly enhancing or hypoechoic masses, retroperitoneal tumors directly invading the kidneys, bilateral renal enlargement, and perirenal soft-tissue masses are suggestive of PRL [5]. However, percutaneous renal biopsy with immunopathological examination is still the gold standard in accurately diagnosing PRL especially in a small lesion as the presentation of renal mass can be similar to renal cell carcinoma and to avoid unnecessary nephrectomy in the PRL patients. Nonetheless, HIV-associated renal cell carcinoma is also rare, the standardized incidence ratio of HIV-associated renal cell carcinoma compared to the general population is only 0.8–2.0, and it is considered as nonacquired immunodeficiency syndrome (non-AIDS) defining the tumor [6].

To our knowledge, the reported cases of primary renal lymphoma in the HIV individual are scarce, and it is one of the AIDS-defining malignancies [7]. Even the incidence of isolated renal lymphoma (extrahemopoietic) in the non-immunocompromised patients is also extremely rare. It occurs only in 0.7% of extranodal lymphoma [8]. The exact cause of PRL is still not well understood as there is no lymphatic tissue in the kidney. It is postulated that PRL originates from the renal capsule and infiltrates the renal parenchyma or chronic inflammation of the kidney causing infiltration of lymphoid cells and transformed into lymphoma [3].

In the HIV patient, lymphomagenesis can happen due to chronic B-cell activation secondary to the immunodeficient state and proliferation of oncogenic viruses (Epstein–Barr virus and Kaposi's sarcoma herpesvirus) and impaired immune control of tumor suppression gene and host genetic alterations. However, despite the early commencement of combined antiretroviral therapy with immune reconstitution, there is no decrease in the incidence of lymphoma among HIV individuals. There are some recent data to suggest that there is a human immunodeficiency virus- (HIV-) encoded protein p17 gene sequences that directly contribute to lymphomagenesis, and strategies to target p17 proteins in the treatment of lymphoma can be beneficial if this association is established [9].

In lymphoma patients with initial presentation of acute kidney injury (AKI), the assessment has to be based on prerenal, renal, and postrenal causes. Volume depletion secondary to poor oral intake and gastrointestinal loss is the commonest cause of prerenal AKI. Intrarenal cause of acute kidney injury in lymphoma can be due to ischemic or nonischemic acute tubular necrosis, direct tumor infiltrates, and renovascular disorder due to the prothrombotic state associated with malignancy or glomerulopathy. However, postrenal causes can occur from direct tumor or lymph nodes compression, and the patient usually presents with oliguric AKI.

AKI primarily due to lymphomatous infiltration is rare and is seen in less than 1% of hematological malignancies [4]. The association is likely due to lymphocytic infiltration to the renal parenchyma resulting in tubular compression and glomerular invasion and impedes blood supply to the nephrons [7]. As the kidneys is not an lymphoid organ, the lymphomagenesis likely originates from the outer surface (cortex) and then invades all the renal structure causing bilateral diffuse lymphocytic infiltration thus damaging the kidneys [10]. As in this case, when he presented to us, the kidneys were massively enlarged and he already had AKI at presentation. His renal biopsy was consistent with lymphomatous infiltration with no normal glomeruli.

However, in the HIV patients treated with tenofovir, nephrotoxicity secondary to tenofovir needs to be considered when the patient presented with acute kidney injury. A review article reported that tenofovir cytotoxicity happens at the mitochondria of the proximal tubule and causes Fanconi syndrome and acute-on-chronic tubulointerstitial nephritis [11]. Thus, closed monitoring of the renal function is important especially in high risk patients with concomitantly taking other nephrotoxic drugs, low CD4 count, and diabetic and hypertension comorbid. Usually, the kidney function improved after withdrawal of the medication.

As described, the likelihood of AKI in this case is due to direct lymphomatous infiltration to the kidney parenchyma as the patient presented with gross hematuria and bilateral massive kidney enlargement which was confirmed by renal biopsy and also PET scan. However, tenofovir toxicity and concomitant renal calculi cause need to be taken into consideration as well because there was an increment of serum creatinine from 108 *µ*mol/L to 193 *µ*mol/L after 12 months of starting tenofovir. The limitation is no sufficient urinalysis and imaging to look for any early lymphomatous infiltrates at that point of time.

There is still no data for prognosis of PRL in HIV individuals. In a study that analyzes the efficacy of R-CHOP in HIV-positive diffuse large B cell lymphoma (DLBCL) and HIV-negative DLBCL, there is no difference in the outcome of both groups of patients [12]. Thus, the standard of treatment of DLBCL regardless of the HIV status is still 6 to 8 cycles of the CHOP regimen with rituximab (monoclonal antibody) in the CD 20 positive patients. It is reported that renal function was markedly improved after giving R-CHOP chemotherapy as the lymphomatous mass shrunk and patient who was dialysis dependent initially was dialysis free [7, 13]. Unfortunately, in this case, he succumbed to death prior to his treatment.

In conclusion, PRL is rare, and it is still one of the differential diagnoses for consideration when a patient presented with renal mass and AKI. The gold standard to confirm the diagnosis is by doing renal biopsy in order to get histopathological sample for examination together with BMAT and PET-CT for staging of the disease. Currently, the standard treatment of PRL is still R-CHOP. Generally, the prognosis of PRL is based on the stage of disease upon diagnosis.

## References

[1] Coggins C. H. (1980). Renal failure in lymphoma. *Kidney international*.

[2] Shetty S., Singh A. C., Babu V. (2016). Primary renal lymphoma-a case report and review of literature. *Journal of Clinical and Diagnostic Research*.

[3] Chen X., Hu D., Fang L. (2016). Primary renal lymphoma: a case report and literature review. *Oncology Letters*.

[4] Luciano R. L., Brewster U. C. (2014). Kidney involvement in leukemia and lymphoma. *Advances in Chronic Kidney Disease*.

[5] Sheth S., Ali S., Fishman E. (2006). Imaging of renal lymphoma: patterns of disease with pathologic correlation. *Radiographics*.

[6] Grulich A. E., van Leeuwen M. T., Falster M. O., Vajdic C. M. (2007). Incidence of cancers in people with HIV/AIDS compared with immunosuppressed transplant recipients: a meta-analysis. *The Lancet*.

[7] Hughes D. J., Fitzgerald N., Sran H. (2014). Acute kidney injury as a presentation of primary renal diffuse large B-cell lymphoma in HIV. *International Journal of STD and AIDS*.

[8] Qiu L., Unger P. D., Dillon R. W., Strauchen J. A. (2006). Low-grade mucosa-associated lymphoid tissue lymphoma involving the kidney: report of 3 cases and review of the literature. *Archives of Pathology and Laboratory Medicine*.

[9] Dolcetti R., Gloghini A., Caruso A., Carbone A. (2016). A lymphomagenic role for HIV beyond immune suppression?. *Blood*.

[10] Obrador G. T., Price B., O’Meara Y., Salant D. J. (1997). Acute renal failure due to lymphomatous infiltration of the kidneys. *Journal of the American Society of Nephrology*.

[11] Fernandez-Fernandez B., Montoya-Ferrer A., Sanz A. B. (2011). Tenofovir nephrotoxicity: 2011 update. *AIDS Research and Treatment*.

[12] Coutinho R., Pria A. D., Gandhi S. (2014). HIV status does not impair the outcome of patients diagnosed with diffuse large B-cell lymphoma treated with R-CHOP in the cART era. *AIDS*.

[13] Erdogmus S., Akturk S., Kendi Celebi Z. (2016). Diffuse large B-cell lymphoma presenting with bilateral renal masses and hematuria: a case report. *Turkish Journal of Haematology*.

